# Social Media Influence on Body Image and Cosmetic Surgery Considerations: A Systematic Review

**DOI:** 10.7759/cureus.65626

**Published:** 2024-07-29

**Authors:** Andreea Mironica, Codruța Alina Popescu, Delaca George, Ana Maria Tegzeșiu, Claudia Diana Gherman

**Affiliations:** 1 Surgery, "Iuliu Hațieganu’’ University of Medicine and Pharmacy, Cluj-Napoca, ROU; 2 Human Sciences, "Iuliu Hațieganu’’ University of Medicine and Pharmacy, Cluj-Napoca, ROU; 3 Thoracic Surgery, Spitalul Clinic de Pneumoftiziologie Leon Daniello, Cluj-Napoca, ROU; 4 Clinical Psychology, Counseling Center for Students, "Iuliu Hațieganu’’ University of Medicine and Pharmacy, Cluj-Napoca, ROU; 5 Surgery-Practical Abilities, "Iuliu Hațieganu’’ University of Medicine and Pharmacy, Cluj-Napoca, ROU

**Keywords:** plastic surgeons, aesthetic treatment, and body image dissatisfaction, social media, cosmetic surgery

## Abstract

Social media platforms like Instagram (Meta Platforms, Inc., Menlo Park, California, United States) and Snapchat* *(Snap Inc., California, United States) significantly influence motivations for aesthetic surgery by promoting idealized and digitally enhanced images. Understanding their impact on body image dissatisfaction and acceptance of cosmetic procedures is crucial. A systematic review following Preferred Reporting Items for Systematic Reviews and Meta-Analyses (PRISMA) guidelines explored the link between social media, body image dissatisfaction, and cosmetic surgery. The review included 25 studies with 13,731 participants. Specific findings revealed that 70% of young adult women and 60% of young adult men report dissatisfaction with their bodies, leading to increased surgical considerations. The search process utilized databases such as PubMed, ScienceDirect, and Google Scholar, employing keywords like "cosmetic surgery," "social media," and "body image dissatisfaction" for articles published between January 2013 and December 2023. Both men and women show increased dissatisfaction with body parts, leading to surgical considerations. Social media's emphasis on visual aesthetics fosters body dissatisfaction and social appearance anxiety, especially through selfies. Cultural norms and celebrity influence further shape beauty perceptions. While social media promotes cosmetic surgery acceptance, ethical concerns about misleading advertisements, unrealistic beauty standards, and patient privacy persist. This underscores the need for strategies to promote healthy body image and informed choices in the digital age.

## Introduction and background

Social media interaction plays a crucial role in shaping individuals' perspectives and motivations for pursuing aesthetic surgery. As platforms like Instagram (Meta Platforms, Inc., Menlo Park, California, United States) and Snapchat (Snap Inc., California, United States) continue to influence societal norms and beauty standards, understanding their effects on body image dissatisfaction (a person's negative perception and feelings about their own body, particularly its appearance) and the acceptance of cosmetic procedures become increasingly important. Despite the growing body of research, there remain specific gaps in the literature, particularly regarding the nuanced ways in which different demographics are affected and the psychological mechanisms underlying these influences. This review aims to fill these gaps by systematically exploring how social media contributes to the desire for cosmetic surgery (a branch of plastic surgery that involves elective procedures aimed at enhancing or altering a person's appearance) across various populations [1,2,3].

Social media has become a primary channel for social communication, offering users opportunities to engage actively with appearance-related content from peers and celebrities. Unlike traditional media, these platforms allow for dynamic interaction, comparison, and discussion, which can significantly impact body image and interest in cosmetic surgery. Recent research indicates that increased exposure to cosmetically enhanced images on social media can elevate an individual's desire for cosmetic procedures [4,5].

Body dissatisfaction and social appearance anxiety (the fear or concern about how one's appearance is evaluated by others in social situations) are significant factors driving the decision to undergo physical modifications. Psychological and sociocultural influences affect how young adults perceive and feel satisfied with their body shape and size, with no specific gender, age, ethnicity, or status being immune to these effects. Studies show that both men and women exhibit increasing discontentment with certain body parts, leading to considerations for cosmetic surgery [1,6-8].

The practice of taking and editing selfies, particularly among younger demographics, further exacerbates body dissatisfaction. Regular engagement in such activities can lead to increased focus on body shape, lower self-esteem, and, in severe cases, contribute to the development of body dysmorphic disorder, a psychiatric condition characterized by an obsessive concern over perceived flaws in appearance [9-11].

Given the prevalence of idealized bodies on social media and the availability of photo editing tools, individuals are often motivated to seek cosmetic surgery to achieve an appearance closer to their edited images. This trend highlights the mutual reinforcement between social media use and body image concerns, with significant implications for psychosocial well-being and happiness [12].

For more detailed definitions and distinctions of different social media platforms and their specific impacts, we can delve into the unique features and influences of Instagram and Snapchat in contrast to traditional media. For example, Instagram's focus on photo sharing and the extensive use of filters can establish high beauty standards, whereas Snapchat's ephemeral content fosters different types of social interactions and pressures. By comparing these platforms to traditional media, such as television and magazines, we can highlight the distinctive ways in which social media's interactive and user-generated content uniquely affects body image and cosmetic surgery considerations.

Understanding the connection between social media, body image dissatisfaction, and the consideration of cosmetic surgery across various demographics offers valuable insights into the decision-making processes for aesthetic procedures. This research highlights the importance for parents, clinicians, and policymakers to acknowledge social media's influence on body image dissatisfaction and the desire for cosmetic enhancements. Additionally, it calls for strategies to promote a healthy body image and self-acceptance in the digital age. The study aims to clarify these relationships to enhance discussions between patients and physicians regarding surgical outcomes and expectations.

## Review

Materials and methods

Study Protocol

The systematic review followed the Preferred Reporting Items for Systematic Reviews and Meta-Analyses (PRISMA) guidelines to identify relevant studies on the link between social media, body image dissatisfaction, and cosmetic surgery consideration.

Inclusion and Exclusion Criteria

The inclusion criteria were studies that identified one or more potential predictive factors of social media on body image dissatisfaction and attitudes toward cosmetic surgery. In this systematic review, studies that focused solely on individuals with body dysmorphic disorder or studies that did not directly assess the relationship between social media and body image dissatisfaction and/or attitudes towards cosmetic surgery and studies where full text was not available were excluded. Articles such as conference abstracts, editorials, and articles not written in English were also excluded.

Search Strategy

In our study, we conducted a comprehensive search across three major databases: PubMed, Science Direct, and Google Scholar. We specifically searched for English-language full-text articles published from 1 January 2013 to 1 December 2023. The search terms included the Mesh term "aesthetic surgery" and the keywords "cosmetic surgery," "social media," and "body image dissatisfaction." Additionally, we employed a combination of Boolean operators to refine our search further. 

**Figure 1:**
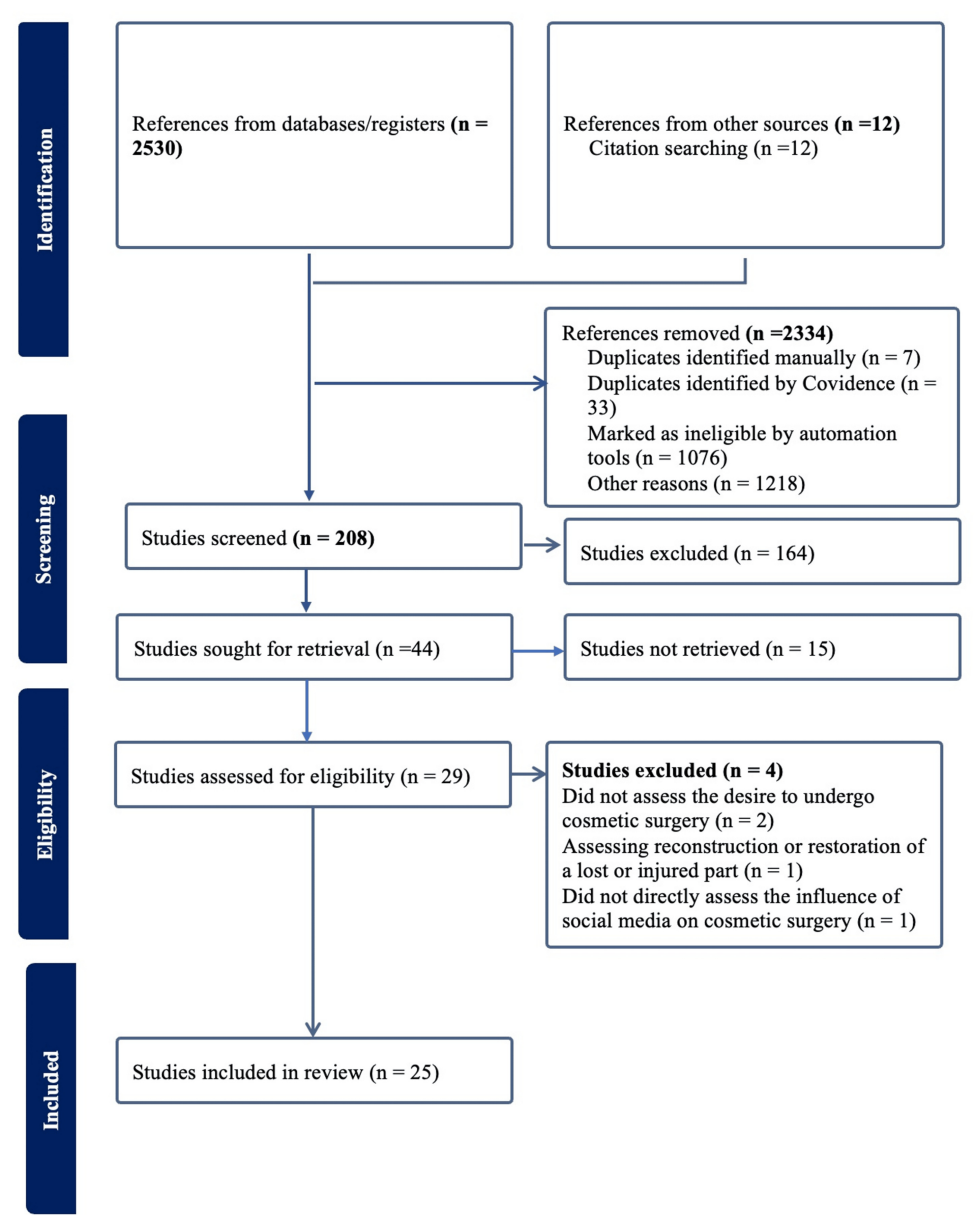
PRISMA flow diagram PRISMA: Preferred Reporting Items for Systematic Reviews and Meta-Analyses

The search strategy is presented in Figure 1.

Quality Assessment

Two reviewers conducted an assessment of study quality using the criteria outlined, and any discrepancies were addressed through discussion. To ensure a robust quality assessment, we utilized specific criteria, including study design, sample size, assessment tools, and the clarity of reported results. Each study was scored based on these criteria to evaluate its methodological rigor and relevance to our review. Each study was individually assessed across four categories: sampling technique, predictive factors, and analysis. Studies that did not meet the preset criteria in these categories were omitted when drawing conclusions.

Data Extraction

Two reviewers independently extracted data from the studies using a standard template, including sample size, study design, participant characteristics, assessment scale, and predictor variables related to body satisfaction and acceptance.

Results

This review included 25 studies with a total of 13731 participants; more details about demographics are presented in Table 1.

**Table 1:** Summary of studies reviewed BMI: body max index; Yo: years old; UK: United Kingdom; SM: social media; SNS: social networking sites

First author and year	Design and aim of the studies	Sample size and demographics	Attitudes, acceptance, interests, perceptions, and beliefs	Considerations, practices, and intentions	Salient findings
Guizzo 2021 [13]	Experimental randomized parallel group; the study aims to investigate the effects of exposure to sexualized imagery and appearance-related comments on Instagram on young women's body satisfaction and intentions to undergo cosmetic surgery.	247 Italian young females; mean BMI 22.00; mean age 23.36 years; 38% Bachelor degree students 61%.	The study demonstrates that Instagram sexualization leads to increased body dissatisfaction among young females, indicating a negative attitude toward body image influenced by social media.	The study demonstrates that Instagram sexualization leads to increased body dissatisfaction among young females, indicating a negative attitude toward body image influenced by social media.	Practical interventions, such as media literacy education and body-positive content, can mitigate the negative impacts of Instagram on body image.
Alghamdi 2020 [14]	Cross-sectional study; aimed to evaluate the attitudes and practices related to cosmetic surgery among university students in Saudi Arabia.	524 Saudi participants; 56.9% females; and 61.6% Bachelor's degree.	There is significant interest in social media advertising and recommendations from celebrities and bloggers.	The intention is to use Instagram and Snapchat as effective marketing tools, with a focus on influencer marketing to engage young consumers.	Social media platforms facilitate active engagement between organizations and consumers, enhancing satisfaction and influencing purchasing decisions.
Ashikali 2017 [6]	Experimental study; aimed to investigate the impact of cosmetic surgery advertising on Swiss women's body image and attitudes towards cosmetic surgery.	145 women, mean age 23.07; 79.3% white; mean BMI was 21.00: 9% underweight, 85.5% normal weight, 5.5% overweight.	Women with materialistic values perceive the intrapersonal benefits of cosmetic surgery differently depending on the nature of the advertisement they are exposed to.	Women low in appearance-related self-discrepancies are more likely to consider surgery when exposed to risk information, highlighting the role of personal dissatisfaction and risk perception in surgical considerations.	The study underscores the influence of the media environment on attitudes towards cosmetic surgery, suggesting that the frequency and style of advertising in different countries (UK vs. Switzerland) could affect how women respond to such ads.
Conboy 2023 [4]	Cross-sectional study; aimed to evaluate the effect of different social media (SM) platforms on plastic cosmetic surgery in Saudi Arabia.	107 women; aged between 18-29, 99% White.	There is a reported high acceptance of cosmetic surgery within the study sample, with a notable interest in dermal fillers, highlighting a trend influenced possibly by celebrity culture and media exposure.	The study reveals that components of self-compassion, particularly over-identification, play a critical role in predicting attitudes toward cosmetic surgery.	Over-identification, a component of self-compassion, was found to significantly predict positive attitudes towards cosmetic surgery, suggesting a nuanced interplay between self-awareness and media influence.
Truasheim 2023 [8]	Cross-sectional study; aimed to explore whether social media engagement impacts men's interest in undergoing cosmetic surgery.	311 participants	The study suggests that the way social media influences men's body image and cosmetic surgery considerations might be unique compared to its influence on women.	The study suggests that the way social media influences men's body image and cosmetic surgery considerations might be unique compared to its influence on women.	The initial hypothesis that active social media engagement would predict cosmetic surgery interest was not supported; instead, passive engagement showed a significant relation.
Zhou 2023 [15]	Cross-sectional quantitative study; the study aimed to explore the influence of social appearance anxiety on social media use, impulsive consumption, and acceptance of cosmetic surgery.	275 responses; mean age 20.91 years; Men’s BMI was 22.34, higher than women's BMI (20.36).	People have a desire to change the perceived weaknesses in their physical appearance due to low body confidence. Social appearance anxiety influences aceptance of cosmetic surgery. Perception that cosmetic surgery can improve emotional well-being, despite potential physical and emotional risks.	Emotional problems such as depression, anxiety, and personality disorders associated with cosmetic surgery. Intention to consume beauty products with the expectation of improved appearance.	Individuals with higher social appearance anxiety engage more in selfie-related behaviors and editing, leading to greater cosmetic surgery intention.
Nerini 2023 [7]	Cross-sectional design; the primary aim of this study was to examine the combined roles of mass media, significant others, and self-awareness (both public and private) in predicting body dissatisfaction and the acceptance of cosmetic surgery for social reasons among young men.	203 Italian men aged 18-35 years with an average BMI of 22.79.	Direct association between media portrayals and men’s body dissatisfaction, but not cosmetic surgery acceptance, suggesting men respond directly to media images without internalizing beauty ideals.	Public self-awareness enhances body dissatisfaction through the internalization of muscularity standards but does not influence the acceptance of cosmetic surgery. Private self-awareness is associated with reduced body dissatisfaction and a lower likelihood of accepting cosmetic surgery for social reasons.	The study notes the absence of social media's role in influencing attitudes toward cosmetic surgery among men, which contrasts with other research suggesting social media's significant impact.
Mortada 2023 [16]	Descriptive cross-sectional survey; the primary aim of the study was to evaluate the use of social media among Saudi plastic surgeons and its influence on their private practices.	61 surgeons; 88.5% were male.	Surgeons believe that social media presence influences patient’s decisions to undergo plastic surgery, with a majority thinking that exposure to plastic surgery on social media increases the likelihood of patients choosing to have procedures.	Social media is increasingly being incorporated into the professional lives of plastic surgeons for marketing and branding purposes, with significant usage among those in private practice (70.6%).	The study found no significant differences in social media use among surgeons based on age, gender, region of residence, or years of practice, suggesting broad acceptance across diverse demographic groups.
Obeid 2022 [17]	Cross-sectional survey; the study aimed to assess how social media influences the decision to undergo rhinoplasty among Saudi patients, with a focus on the most used social media platforms and the impact of advertisements and before-and-after photos.	205 participants 54.1% were single; 61.5% were employed; 91.2% were women; and had a Bachelor’s degree (80.5%).	A significant percentage of participants acknowledged the impact of social media on their decision to undergo rhinoplasty, highlighting a broad acceptance and interest in aesthetic enhancements influenced by digital platforms.	Platforms like Snapchat and Instagram are commonly used among the participants, with Snapchat being particularly popular due to its privacy features.	The visual impact of before and after images was a dominant factor, affecting 76.1% of the patients, indicating a significant visual-driven decision-making process in cosmetic surgery considerations.
Taha 2023 [18]	Cross-sectional survey; the study aimed to investigate the influence of social media on the decision to undergo cosmetic procedures.	With 1654 participants; Saudi nationals (96.80%). Female 69.11%, age group of 18-30 years 64.27%; bachelor’s degree 68.74%.	A significant portion of participants (28.42%) reported being influenced by social media to consider rhinoplasty, primarily driven by images of celebrities before and after such procedures.	A majority of respondents (64.69%) found information about rhinoplasty predominantly on Snapchat, highlighting the platform's role in shaping cosmetic surgery considerations.	The desire to improve appearance for selfies and video conferencing, intensified by the COVID-19 pandemic, has significantly increased the consideration for facial cosmetic procedures, particularly among those dissatisfied with their facial features.
AlBahlal 2023 [1]	Cross-sectional study; aimed to evaluate the effect of different social media (SM) platforms on plastic cosmetic surgery in Saudi Arabia.	2248 participants; 46.2% of them belonged 21 to 30-year-old group, 71.2% were females, and 91.4% were Saudi citizens.	A substantial portion of participants, influenced by social media, were more interested in cosmetic treatments compared to those who were not influenced by SM, showing a strong relationship (P < .001) between social media influence and the interest in undergoing cosmetic treatments.	Surgeons' advertisements on SM, showing before-and-after pictures, significantly influenced the decisions of participants to seek consultations and treatments, more so among those influenced by SM than those who were not.	The interaction of photo-editing apps and SM exposure significantly correlates with the increased desire for cosmetic surgery, highlighting the impact of visual presentation and aesthetic enhancements in decision-making.
Alghonaim 2019 [19]	Cross-sectional study; to assess the impact of social media on aesthetic procedures among females in Riyadh, Saudi Arabia.	1449 Saudi females; 46.75% were aged between 25 and 34 years; 55.09% were single; 77.29% held a Bachelor’s degree.	Instagram was notably the most influential platform concerning aesthetic procedures, especially among the young adult demographic.	The majority of the participants used social media, alongside family and friends' experiences, as their primary information sources before deciding on aesthetic procedures.	The findings underline an evolving trend where plastic surgeons increasingly leverage social media to enhance visibility, educate potential patients, and build a professional brand.
Alkarzae 2020 [20]	Cross-sectional study; to investigate the impact of social media on cosmetic surgery decisions among women in Saudi Arabia.	653 individuals; mean age 29.4 yo; 25.1% were male and 74.9% were female; 35.8% were married. 71.4% had a Bachelor’s degree.	A significant interest in cosmetic procedures due to dissatisfaction with appearance in selfies. 37.8% of respondents expressed a desire to undergo cosmetic procedures due to selfies, with rhinoplasty being the most desired.	Intentions to undergo cosmetic procedures to correct perceived deformities revealed by selfies, with 46% of those perceiving deformities expressing a desire for surgical correction. Bringing selfies to cosmetic surgery consultations to illustrate perceived deformities.	Increased frequency of encountering one's photographic likeness due to social media, leading to heightened interest in cosmetic surgery.
Arab 2019 [2]	Cross-sectional study; to assess the role of social media in shaping cosmetic surgery decisions among young adults.	816 Saudi participants; mean age 21.15.	A significant number of female university students are influenced by social media in their decision to undergo cosmetic treatments, with nearly half reporting being swayed by advertisements.	Social media advertisements significantly influence the consideration of undergoing cosmetic treatments, with a preference for non-surgical procedures among those affected.	Exposure to cosmetic surgery advertisements and influencer content on social media platforms can affect self-esteem and perceptions of attractiveness, leading to increased interest in cosmetic procedures.
Wang 2022 [21]	Cross-sectional observational analytic study; the study aimed to explore the associations between body talk on social networking sites (SNS), body surveillance, and body shame with the consideration of cosmetic surgery among young adults in China.	309 college students from China;62.5% women and 37.5% men; average age of 18.98 years; mean BMI 20.80.	The study suggests that engagement in discussions about body appearance on social media (SNS body talk) increases personal concern about body image, which in turn increases the consideration of cosmetic surgery.	There is an indirect association between SNS body talk and cosmetic surgery consideration via the mediators of body surveillance and body shame.	The study highlights a cultural shift in China, with increasing acceptance of cosmetic procedures among men, potentially influenced by celebrity culture and possibly Western media ideals; the study did not find any gender differences in how these factors influence cosmetic surgery consideration.
DiGesto 2022 [22]	Cross-sectional study; to investigate the psychological effects of social media on body image among university students.	305 Caucasian Italian university women aged 19–32 years; mean BMI was 21.78; 94.6% were unmarried and 5.4% were married or cohabiting; 97.2% were students and 2.8% workers.	Activities such as taking, selecting, and editing photos are a form of body checking, where women assess and compare their bodies against sociocultural standards, influencing their perceptions and acceptance of cosmetic surgery.	Engagement with Instagram, especially images related to celebrities, directly and indirectly, increases the acceptance of cosmetic surgery through mechanisms like appearance comparison and body dissatisfaction.	Beyond Instagram, other sociocultural and individual factors such as peer influence, family attitudes, and personal beliefs about physical appearance play crucial roles in shaping attitudes towards cosmetic surgery.
Jung 2016 [23]	Descriptive, cross-sectional survey study; the primary aim of the study is to explore the associations between attitudes toward cosmetic surgery, the extent of celebrity worship, and body image dissatisfaction among South Korean and US female college students.	370 female undergraduates, with 196 from a South Korean university and 174 from a university in the mid-Atlantic region of the US. The participants were 86.8% White, 6.3% Asian, and 6.9% from other ethnic backgrounds. The average BMI was 19.56 for Korean participants and 21.74 for US participants.	U.S. participants show a stronger positive attitude towards celebrities than South Korean participants. This positive celebrity attitude is associated with a greater acceptance of cosmetic surgery in the U.S. U.S. participants displayed greater body satisfaction than South Korean participants.	South Koreans show a higher likelihood of accepting cosmetic surgery, possibly due to different societal norms or lesser stigma associated with such procedures.	The influence of celebrities on cosmetic surgery acceptance is more pronounced in the U.S., indicating that celebrity culture might play a larger role in shaping cosmetic surgery attitudes in Western contexts; lower body appreciation significantly predicts a greater acceptance of cosmetic surgery, highlighting body image satisfaction as a crucial factor in cosmetic surgery considerations.
Padley 2022 [10]	Cross-sectional survey; the study aimed to examine the psychological impact of the COVID-19 pandemic on body-image perception and the resulting increase in requests for cosmetic surgery.	The study included 159 respondents, with a gender distribution of 73% females and 27% males. The average age was 43 years, with 56% of participants based in the UK and 44% in Italy.	During the pandemic, patients have shown an increased inclination to undergo cosmetic surgery to enhance their appearance for video conferencing. It is believed that constant exposure to one’s image during video calls can distort self-perception.	Psychological impact and mood levels during the pandemic should be considered as factors influencing the decision to seek cosmetic surgery.	The need for plastic surgeons to assess the psychological motivations behind cosmetic surgery requests and to conduct further studies to ensure patient safety and satisfaction.
Hermans 2022 [5]	Observational analytic study; to examine the impact of social media on body image and cosmetic surgery interest among young women.	470 responses; Dutch young adults between 18-25 yo; 44% males and 55.6% females; 23.6% higher education.	Following influencers who underwent cosmetic procedures does not significantly relate to acceptance, but this might be due to the content of the acceptance scale focusing on self-image benefits. Young adults exhibit a relatively low interest in cosmetic procedures, particularly in treatments less popular among younger demographics, such as Botox.	The more frequently young adults use visual social media like Instagram and TikTok, the more they consider cosmetic procedures. Passive social media use, such as following influencers who had cosmetic procedures, correlates with increased intention and normalization.	Despite high perceived prevalence, actual interest in cosmetic procedures among young adults is low, indicating a discrepancy between perceived and actual popularity.
AlGhadeer 2021 [24]	Cross-sectional study; the study aimed to assess the impact of self-esteem and self-perceived body image on the acceptance of cosmetic surgery among Saudi adults.	1008 responses; mean age of 34.7 yo; 73.5% females; 76.2% were married; 85.3% participants were university graduates.	Higher acceptance among older participants, unmarried groups, and those with chronic health problems. Interest in cosmetic surgery is influenced by factors such as body image orientation, being teased for appearance, and knowing someone who had cosmetic surgery.	Gender and age differences in intentions, with younger respondents showing higher acceptance for social reasons. Differences in practices across countries and cultures, with women comprising a large majority of cosmetic surgery patients. Differences in practices across countries and cultures, with women comprising a large majority of cosmetic surgery patients.	Significant association between high self-esteem and high body appreciation, leading to lower acceptance of cosmetic surgery.
Walker 2021 [25]	Experimental study; the study aimed to examine whether exposure to images depicting facial cosmetic enhancements increases the desire for cosmetic surgery among young women.	The sample consisted of 118 English women with an average age of 20.71 years, comprising 50.9% White, 25.3% Asian, 10.1% Black, and 13.5% from other ethnic backgrounds.	Participants who regularly engage with social media and feel dissatisfied with their appearance are more likely to consider cosmetic surgery in the future.	The inclination to pursue cosmetic surgery was somewhat influenced by viewing cosmetically enhanced images versus travel images.	Social media plays a more substantial role in influencing the desire for cosmetic surgery compared to body dissatisfaction, challenging some previous assumptions.
Yahya 2020 [26]	Cross-sectional study; the study aimed to investigate the impact of social media engagement on body image and the increased popularity of seeking cosmetic surgery in Al-Ahsa City, Saudi Arabia.	513 participants; mean age 28.54 yo; 90.1% were females; 57.1% were married; 69.9% were university students or graduates.	Social media is accepted as a significant influencer on physical appearance and cosmetic surgery decisions.	The time spent on social media, especially 15 minutes before sleeping and after waking up, is a critical factor influencing cosmetic surgery decisions. Intention to change body image to align with an ideal influenced by social media.	An increase in cosmetic surgeries from 17.2% in 2014 to 18.2% in 2017, parallel with the rise in social media engagement.
Sindi 2023 [27]	Cross-sectional survey; the study aimed to evaluate the effect of social media platforms on the decision-making process of the general population regarding cosmetic procedures.	364 participants; mean age 27.4; mean BMI of 25.0; 60% were female; more than half are undergraduates; most of the participants were Saudis.	Despite high social media usage, most participants had no intentions to undergo cosmetic procedures, contrasting with previous literature. Makkah society's conservative nature may affect attitudes towards social media and cosmetic procedures.	Participants who spent less time on WhatsApp and Twitter were less likely to consider future cosmetic procedures, indicating platform-specific influences. The most popular cosmetic procedures in the studied population include dermal fillers and rhinoplasty.	Internal and external factors, such as emotional states and societal pressures, play a role in the decision to undergo cosmetic procedures. The type of social media platform influences the acceptance and consideration of cosmetic procedures.
Alhusaini 2022 [28]	Cross-sectional survey; aimed to investigate the impact of Snapchat use on self-image and the inclination toward cosmetic procedures.	1,064 participants; 41.1% were less than 25 yo; 82.1% were females; 74.9% were university degree holders; 49.1% single.	80% of the population showed interest in undergoing cosmetic procedures, influenced by social media. Interest is driven by goals such as increasing social media followers and filtering self-image on platforms like Snapchat.	Increased willingness to undergo cosmetic procedures due to social media influence and negative self-views while using social media. The potential complications post-surgery and their impact on the patient's quality of life are significant considerations.	There is a notable discrepancy between the perceived benefits and the reliability of the information provided by social media regarding cosmetic procedures. The inclination towards cosmetic surgery is higher among older participants, females, educated individuals, married individuals, and those with previous cosmetic surgery history.
Jieh 2022 [12]	Quantitative study using purposive sampling; to investigate the relationships between selfie-editing behavior, self-esteem, and social appearance anxiety among university students in Malaysia.	253 students; 56 male and 195 female; 204 undergraduate; 172 between 21-25 yo.	The text suggests that frequent selfie-editing is perceived negatively, as it is associated with increased social appearance anxiety and lower self-esteem. This implies an attitude that values natural appearance over edited selfies. Students perceive a significant relationship between selfie-editing and their social and psychological health. The belief that selfie-editing leads to negative outcomes suggests a perception that the practice is detrimental.	The intention behind the study is to explore and establish the correlation between selfie-editing, social appearance anxiety, and self-esteem. There is also an intention to compare these findings with previous studies to identify consistency or discrepancies.	A weak and negative correlation was found between the Photo Manipulation scale and self-esteem. Selfie-editing prior to online posting significantly influenced social appearance anxiety, accounting for 5.5% of the variance. The results contradict a past experiment suggesting that selfie-editing reduces social anxiety. This discrepancy may be due to differences in the focus on immediate versus long-term effects of selfie-editing.

Factors Associated with Likeliness to Pursue Cosmetic Surgical Procedure

Social media influence: The pervasive nature of social media platforms such as Instagram and Snapchat plays a crucial role in shaping individuals' decisions to undergo cosmetic procedures. These platforms are highly visual, emphasizing appearance through images and videos. Users are constantly exposed to idealized and often digitally enhanced images, setting high and sometimes unrealistic beauty standards. The constant comparison with these images can lead to dissatisfaction with one's appearance. The engaging and interactive features of social media, including likes, comments, and shares, further reinforce the desire to conform to these standards, driving individuals to consider cosmetic enhancements to align with the prevalent aesthetic ideals [5,14,17,18,19].

Selfie behavior: The behavior of frequently taking and editing selfies has become a common trend, particularly among younger demographics. This practice is linked to higher considerations for cosmetic surgery. The process of taking selfies often involves scrutinizing one’s appearance closely, identifying perceived flaws, and using filters or editing tools to "improve" these features. Over time, this can lead to increased body dissatisfaction as individuals become more aware and critical of their imperfections. The desire to look better in selfies can translate into a willingness to undergo cosmetic procedures to achieve a more polished and attractive appearance that can be effortlessly captured on camera [4,15,20].

Body dissatisfaction and anxiety: Anxiety over social appearance and body dissatisfaction are significant predictors of the desire to undergo cosmetic surgery. Individuals who are unhappy with their bodies are more likely to seek surgical solutions to improve their self-image. Social appearance anxiety, which involves the fear of negative evaluation of one’s appearance by others, exacerbates this desire. The pressure to meet societal beauty standards can lead to chronic anxiety and dissatisfaction, prompting individuals to consider cosmetic surgery as a way to alleviate these feelings and improve their confidence and social acceptance [2,7,15].

Cultural and societal norms: These factors heavily influence the perception of beauty and the acceptance of cosmetic procedures. In regions with high exposure to Western beauty standards, there is often a greater emphasis on certain aesthetic ideals, such as slim bodies, symmetrical facial features, and youthful appearances. These norms are propagated through various media, including movies, advertisements, and social media, creating a widespread belief in the desirability of these features. The pressure to conform to these standards can be immense, leading individuals to seek cosmetic surgery to fit the cultural and societal mold of beauty [21,23].

Celebrity influence: Celebrities play a significant role in shaping public perceptions of beauty and cosmetic surgery. Their endorsements and visible transformations are highly influential, particularly on social media. When celebrities undergo cosmetic procedures and publicly share their experiences and results, they normalize these practices and make them more desirable to the public. Followers who admire these celebrities may be motivated to undergo similar procedures to emulate their idols. The portrayal of flawless celebrity appearances as achievable goals can significantly sway individuals' decisions to pursue cosmetic surgery, viewing it as a legitimate and accessible means to enhance their own looks [5,22,25].

Positive Attitudes Toward Cosmetic Surgical Procedure

Enhanced appearance: Many individuals view cosmetic procedures to improve their physical appearance. This perspective is rooted in the desire to address specific aesthetic concerns that may not be achievable through natural means. Whether it’s correcting perceived imperfections, reversing signs of aging, or enhancing certain features, cosmetic surgery provides a solution to attain desired looks. This enhancement can lead to increased self-esteem and confidence, as individuals feel more satisfied with their appearance. The notion that looking better can lead to feeling better is a significant driver behind the positive attitudes towards cosmetic procedures [1,2,20].

Social media validation: In the age of social media, achieving a certain look for online approval is seen as a benefit of cosmetic surgery. Platforms like Instagram and Snapchat place a high value on visual aesthetics, and users often seek validation through likes, comments, and shares. Cosmetic procedures can help individuals conform to the beauty standards prevalent on these platforms, thus enhancing their social media presence. The positive feedback received from followers can further boost self-esteem and provide a sense of social acceptance. For many, the ability to present an idealized version of themselves online is a compelling reason to undergo cosmetic enhancements [5,14,17-19].

Emotional well-being: There is a belief that cosmetic surgery can lead to better emotional well-being despite the potential risks involved. For individuals who struggle with body image issues or have long-standing insecurities about their appearance, cosmetic procedures offer a way to alleviate these concerns. The satisfaction derived from seeing a more desirable reflection in the mirror can translate to improved mental health, reducing feelings of depression and anxiety related to body dissatisfaction. This improvement in emotional well-being is often cited by proponents as a significant benefit of cosmetic surgery [10,15].

Negative Attitudes Toward Cosmetic Surgical Procedure

Risk awareness: Awareness of the potential physical and emotional risks associated with cosmetic surgery is a major factor contributing to negative attitudes. Complications from surgery, such as infections, scarring, and adverse reactions to anesthesia can deter individuals from pursuing these procedures. Moreover, the results of cosmetic surgery may not always meet expectations, leading to dissatisfaction and regret. The possibility of facing unmet expectations and the associated disappointment is a significant concern that fosters skepticism towards cosmetic procedures [6,28].

Body image issues: Concerns about fostering negative body image and unrealistic beauty standards are prevalent among critics of cosmetic surgery. The pursuit of an idealized appearance can perpetuate a cycle of dissatisfaction, where individuals continually find new flaws to fix. This focus on physical perfection can detract from the appreciation of natural beauty and individuality. Additionally, the promotion of certain beauty standards through cosmetic surgery can lead to societal pressures, making others feel inadequate if they do not conform. These issues highlight the potential negative impact of cosmetic procedures on overall body image [6,15,20].

Psychological impact: Elevated rates of depression, anxiety, and personality disorders are observed among those considering cosmetic surgery. The desire to undergo these procedures often stems from deeper psychological issues related to self-worth and identity. For some, the temporary boost in self-esteem provided by cosmetic enhancements may not address the underlying causes of their dissatisfaction. Instead, it can lead to a dependence on surgical solutions to manage emotional distress. The psychological toll of cosmetic surgery, including the potential for post-operative regret and the need for further procedures, contributes to the negative attitudes surrounding these interventions [10,15].

Acceptance of Cosmetic Surgical Procedure

High acceptance: Cosmetic surgery enjoys a high acceptance rate, particularly among younger demographics and active social media users. This trend can be attributed to several factors. Firstly, the youth are generally more receptive to new trends and are significantly influenced by the visual culture of social media. Platforms like Instagram and TikTok (ByteDance Ltd., Beijing, China) propagate idealized images of beauty, often enhanced by cosmetic procedures. Young people, exposed to these standards daily, may seek cosmetic surgery to align their appearance with these ideals. Moreover, social media fosters a culture of constant self-presentation and comparison, which can intensify desires to meet specific aesthetic standards. The validation received through likes and positive comments post-surgery further reinforces the decision to undergo cosmetic enhancements [5,24].

Cultural differences: Acceptance of cosmetic surgery varies significantly across different cultures, influenced by societal norms and the level of stigma associated with such procedures. In some cultures, cosmetic surgery is seen as a routine and acceptable means of improving one’s appearance. For example, South Korea has one of the highest frequencies of cosmetic surgery procedures globally, with procedures deeply embedded in societal norms and widely accepted. Conversely, in more conservative societies, cosmetic surgery may be less common and more stigmatized. However, globalization and the proliferation of Western beauty ideals through media have led to increasing acceptance in many regions traditionally resistant to cosmetic enhancements. The cultural context thus plays a crucial role in shaping attitudes toward cosmetic surgery [21,23,27].

Influence of education and marital status: Acceptance rates of cosmetic surgery are also higher among individuals with higher education levels and those in different marital statuses. Education often correlates with greater awareness and understanding of cosmetic procedures, including the potential benefits and risks. Individuals with higher education often have greater access to information and resources, allowing them to make well-informed decisions regarding surgery. Additionally, marital status influences acceptance, with single individuals and those recently divorced showing higher rates of acceptance. These groups may be more motivated to enhance their appearance, either to boost self-confidence or to attract new partners. The interplay between education, marital status, and the desire for cosmetic surgery underscores the complexity of factors driving acceptance [17,26,28].

Perception of Others Who Undergo Cosmetic Surgical Procedure

Admiration for improvement: Individuals who undergo cosmetic procedures often receive admiration for their enhanced appearance. The visible improvements resulting from these procedures can elicit positive reactions from peers, family, and society at large. This admiration is rooted in the effort and willingness to invest in one's appearance, which is often seen as a form of self-care and personal enhancement. The post-surgery results, whether subtle or dramatic, are typically viewed as successful attempts to achieve aesthetic goals. This positive feedback can reinforce the decision to undergo cosmetic surgery, contributing to a cycle of approval and increased self-esteem for those who have opted for such procedures [19,22].

Celebrity influence: The influence of celebrities and social media influencers who have undergone cosmetic procedures significantly shapes public perceptions. When admired public figures share their cosmetic surgery experiences and results, they contribute to normalizing these practices. Followers who look up to these celebrities may develop positive views towards cosmetic surgery, seeing it as an acceptable and even desirable means of enhancing one's appearance. This normalization is amplified by the transparency and openness with which many celebrities discuss their procedures, often framing them as empowering choices that align with personal or professional aspirations. The portrayal of cosmetic surgery by influencers can thus foster a more accepting and positive attitude towards these enhancements among their audiences [6,23].

Superficial judgments: Despite the positive admiration some receive, there is a prevalent negative perception that individuals who undergo cosmetic surgery are superficial or overly concerned with their appearance. This judgment stems from the belief that altering one's appearance through surgery indicates a prioritization of looks over more substantive personal qualities. Critics argue that such individuals may be focusing too much on external validation rather than internal growth or self-acceptance. This superficial label can carry a stigma, suggesting that those who choose cosmetic surgery are more interested in vanity than authenticity, potentially leading to societal disapproval or ridicule [22].

Unrealistic standards: Another significant negative perception is that cosmetic surgery promotes unrealistic beauty standards. The enhanced and often idealized results of cosmetic procedures can set unattainable benchmarks for beauty, leading to widespread body dissatisfaction. This issue is exacerbated by the portrayal of post-surgery appearances in media and advertising, which often highlight flawless and exaggerated outcomes. Critics argue that these representations contribute to a culture that values a narrow and often artificial standard of beauty, pressuring individuals to undergo surgery to conform. This pursuit of perfection can lead to a cycle of continual dissatisfaction and repeated surgical interventions, undermining the appreciation of natural diversity in appearance [1,10,20].

The Surgical Profession

In the contemporary medical landscape, plastic surgeons increasingly leverage social media platforms for marketing, enhancing visibility, and engaging with patients. Platforms such as Instagram, Facebook, and TikTok enable surgeons to showcase their work through before and after photos, videos, and patient testimonials, effectively serving as a dynamic portfolio that highlights their skills and the transformative effects of their procedures. Additionally, social media offers an interactive space where potential patients can ask questions, seek advice, and receive immediate feedback, fostering trust and credibility. This interaction helps patients feel more informed and comfortable in their decision-making process. Surgeons also utilize these platforms to stay updated with industry trends, connect with peers, and participate in professional communities [1,14].

The majority of plastic surgeons believe that social media positively influences patient decisions and significantly enhances the visibility of their field. By using social media, surgeons can reach a broader audience, including individuals who might not have considered cosmetic surgery otherwise. The transparency and accessibility of information on these platforms empower patients to make more informed choices. Additionally, social media allows surgeons to share educational content, debunk myths, and provide insights into the realities of cosmetic surgery. This proactive dissemination of knowledge helps demystify procedures, reduce patient anxiety, and manage expectations. Overall, the consensus among surgeons is that social media is an invaluable asset for their practice, facilitating growth and patient engagement [16].

However, the use of social media by plastic surgeons also raises important ethical considerations. Concerns include the potential for misleading advertisements and the portrayal of unrealistic results, which can lead to patient dissatisfaction and harm. The emphasis on aesthetic perfection propagated through social media can contribute to body image issues and the pursuit of unnecessary surgeries. Ethical promotion requires surgeons to maintain honesty and integrity in their posts, ensuring that the information shared is accurate and balanced. Additionally, there are concerns about patient privacy and consent. Surgeons must obtain proper consent before sharing any patient-related content and respect the confidentiality of patient information [17].

Representation of Cosmetic Surgical Procedure in the Media

Social media platforms: Instagram and Snapchat have emerged as dominant platforms that significantly influence perceptions and decisions regarding cosmetic surgery. These platforms are highly visual, allowing users to share images and videos that emphasize appearance. On Instagram, the culture of aesthetic perfection is prevalent, with users constantly exposed to images of idealized beauty standards. The use of filters and editing tools on Snapchat also contributes to this phenomenon, as users can experiment with altered versions of their appearance. The frequent display of enhanced looks creates a comparative environment where individuals feel pressured to conform to these beauty ideals. The interactive nature of these platforms, where users receive immediate feedback through likes and comments, further reinforces the desire to attain similar results through cosmetic procedures [14,17,18].

Visual impact: The impact of visual content, particularly before and after images, is profound in driving the decision-making process for cosmetic surgery. These images provide tangible proof of the potential transformations that cosmetic procedures can achieve. Seeing dramatic improvements in appearance can be highly persuasive, as it offers a clear and immediate representation of the benefits. Before and after photos allow individuals to visualize the possibilities for their own bodies, making the decision to undergo surgery more compelling. This visual-driven approach appeals to the desire for quick and noticeable results, highlighting the effectiveness of cosmetic interventions. The allure of achieving a similarly impressive transformation motivates many to pursue these procedures [13,16,28].

Advertising: Cosmetic surgery advertisements on social media are pivotal in influencing decisions, with a significant number of participants reporting being swayed by such ads. These advertisements often feature testimonials, endorsements, and visually appealing content that highlights the success of various procedures. Social media algorithms also play a role in targeting potential candidates by showing ads based on users’ interests and behaviors. The persuasive nature of these ads, combined with their frequent appearance in users' feeds, can create a strong incentive to consider cosmetic surgery. Advertisements that focus on the ease, affordability, and accessibility of procedures further reduce the barriers to making a decision. The strategic placement and compelling content of cosmetic surgery ads contribute to their effectiveness in shaping public perceptions and encouraging individuals to seek out these services [1,14,16].

Discussion

The influence of social media platforms like Instagram and Snapchat is significant in shaping individuals' decisions to undergo cosmetic procedures. These platforms emphasize visual aesthetics, exposing users to idealized and often digitally enhanced images, which can lead to dissatisfaction with one's appearance and drive the desire for cosmetic enhancements [12,26,29,30]. The practice of taking and editing selfies, particularly among younger demographics, further heightens body dissatisfaction and increases the consideration for cosmetic surgery [1,16,20].

Body dissatisfaction and social appearance anxiety are major predictors of the desire for cosmetic surgery [11,31]. Individuals unhappy with their bodies or fearing negative evaluation by others are more likely to seek surgical solutions [2,15,32]. Cultural and societal norms, especially those emphasizing Western beauty standards, also play a crucial role in influencing perceptions of beauty and acceptance of cosmetic procedures [27,33,34].

Celebrities and social media influencers significantly shape public perceptions of beauty and cosmetic surgery by normalizing these practices through their endorsements and transformations [4,5,23]. Positive attitudes toward cosmetic surgery include improved physical appearance, increased self-esteem, and enhanced social media validation [1,2,25,35,36]. However, there are negative attitudes due to awareness of potential risks, fostering unrealistic beauty standards, and the psychological impact, including dependence on surgical solutions for emotional distress [10,28].

Cosmetic surgery enjoys high acceptance, especially among younger demographics and social media users, due to the influence of visual culture [24]. Acceptance varies across cultures and is higher among educated individuals and certain marital statuses [17,26,28]. Despite some admiration for improved appearance post-surgery, there are negative perceptions that cosmetic surgery promotes superficiality and unattainable beauty standards [22].

Plastic surgeons increasingly use social media for marketing, showcasing their work, and engaging with patients [37,38]. While most surgeons believe social media positively influences patient decisions and enhances visibility, there are ethical concerns about misleading advertisements and patient privacy [16,34,39]. Misleading advertisements can create unrealistic expectations and lead to patient dissatisfaction when results do not match the portrayed outcomes. Expanding the discussion on these ethical concerns, it is essential to highlight that misleading advertisements not only deceive potential patients but also undermine trust in the medical profession. Surgeons must adhere to high standards of transparency and honesty in their marketing practices. Additionally, patient privacy must be rigorously safeguarded, ensuring that all shared content respects confidentiality agreements and is used with explicit patient consent. Furthermore, patient privacy should be a top priority, with stringent measures in place to protect personal information and maintain the integrity of the patient-doctor relationship. By addressing these ethical issues, the cosmetic surgery industry can promote a more responsible and patient-centered approach, ultimately enhancing the credibility and sustainability of the field. Overall, social media significantly impacts the perception and acceptance of cosmetic surgery [17,18,28,34,40-42].

A more detailed critical analysis reveals several limitations and potential biases in the included studies. Many studies rely on self-reported data, which can introduce response bias. The cross-sectional nature of most research does not establish causality, only correlation. Additionally, the representation of various demographics is often uneven, with a focus on younger, Western populations, limiting the general applicability of the findings.

Potential biases in the review process include publication bias, where studies with significant findings are more likely to be published. Language bias may also be present, as non-English studies were excluded. Future reviews should aim to include a more diverse range of studies to mitigate these biases. Future research should focus on conducting longitudinal studies to establish causality between social media use and body image dissatisfaction. It is also important to include more diverse populations in terms of age, ethnicity, and cultural background to improve the universality of findings.

## Conclusions

Social media platforms like Instagram and Snapchat significantly influence the desire for cosmetic procedures by promoting idealized images, which increase body dissatisfaction and social appearance anxiety. Cultural norms and celebrity influence further shape beauty perceptions, leading to positive attitudes towards improved appearance and negative concerns about risks and unrealistic standards. The high acceptance of cosmetic surgery among younger demographics and social media users is evident. While plastic surgeons effectively use social media for marketing, there are ethical concerns about misleading ads and patient privacy. Overall, social media profoundly impacts cosmetic surgery perceptions, emphasizing the need for informed and safe choices.

To mitigate these negative impacts, clinicians should implement body positivity programs and screen for psychological conditions before cosmetic procedures. Policymakers need to enforce stricter advertising regulations and support mental health programs addressing social media-related body image issues. Social media platforms should promote body positivity, collaborate with mental health professionals, and establish guidelines to limit the promotion of unrealistic beauty standards. These steps can help mitigate the negative impacts of social media on body image and cosmetic surgery considerations, fostering a healthier and more realistic perception of beauty.
